# The supplemental use of infant formula in the context of universal breastfeeding practices in Western Nepal

**DOI:** 10.1186/s12887-016-0602-1

**Published:** 2016-05-21

**Authors:** Vishnu Khanal, Jane A. Scott, Andy H. Lee, Rajendra Karkee, Colin W. Binns

**Affiliations:** Nepal Development Society, Bharatpur, Chitwan Nepal; School of Public Health, Curtin University, Perth, Australia; School of Public Health and Community Medicine, BP Koirala Institute of Health Sciences, Dharan, Nepal

**Keywords:** Breastfeeding, Baby formula, Cohort studies, Infant food, Infant Formula, Nepal, Urban Health

## Abstract

**Background:**

While the initiation of breastfeeding is universal in Nepal, little has been reported on formula feeding practices. This study aimed to report the prevalence of, and factors associated with, the use of infant formula as supplementary feeds in the Western region of Nepal.

**Methods:**

A community-based cohort study was conducted to collect infant feeding information among 735 postpartum mothers using structured questionnaires. Complete formula feeding data were collected from 711 women in the first, fourth and sixth month postpartum. Factors independently associated with formula feeding were investigated using multiple logistic regression.

**Results:**

All mothers were breastfeeding their infants at the time of recruitment. The prevalence of formula feeding was 7.5 % in the first month and 17 % in the sixth month. About a quarter of mothers (23.8 %) reported providing infant formula at least once during the first six months of life. Infant formula was used commonly as top-up food. Stepwise logistic regression showed that infants born to families residing in urban areas (adjusted odds ratio (aOR): 2.14; 95 % confidence interval (CI): 1.37 to 3.33), mothers with higher education (aOR: 2.08; 95 % CI: 1.14 to 3.80), and infants born by caesarean section (aOR: 1.96; 95 % CI: 1.21 to 3.18) were at greater risk of formula feeding.

**Conclusion:**

The current findings indicate that health workers should support mothers to initiate and continue exclusive breastfeeding particularly after caesarean deliveries. Furthermore, urban health programs in Nepal should incorporate breastfeeding programs which discourage the unnecessary use of formula feeding. The marketing of formula milk should be monitored more vigilantly especially in the aftermath of the April 2015 earthquakes or other natural disasters.

## Background

Worldwide sub-optimal breastfeeding is contributing solely to about 13 % of child mortality and 10 % of childhood diseases [1–3] and about 50 per cent of diarrheal episodes could be avoided with optimal breastfeeding practices [4]. The early introduction of supplemental infant formula increases the risk of childhood diarrhoea [3, 5] as formula does not contain the bioactive and immune protective properties of breastmilk [6, 7]. Furthermore, providing infants with infant formula requires powdered formula to be mixed with boiled water under hygienic conditions. Therefore, if formula powder is reconstituted with unsafe water or prepared and fed under unhygienic conditions the risk of infants suffering diarrhoea is increased [3]. In addition, the risk of disease and deaths as a result of infant formula use are higher in developing countries due to low literacy levels and inadequate skills of carers required to prepare infant formula safely [3, 8].

The initiation of breastfeeding is universal in Nepal; however, the use of animal milk and other fluids as supplementary feeds is relatively common [9]. While the Nepal Demographic and Health Survey 2011 reported that only 0.8 % of infants aged <1 month, and 2.6 % aged 6-8 months were provided infant formula [10], a more recent longitudinal study from central Nepal found a much higher prevalence of usage of 1.7 % among infants aged less than 1 month and 13.7 % among infants in their sixth month [9]. Diarrhoea is one of the most frequent illnesses among Nepalese children and infants [11] and the use of infant formula is a likely contributor to the high incidence of diarrhoea which in the years 2010/2011, 2011/2012 and 2012/2013 was 500, 528 and 578 per 1000 under five-year children, respectively [10].

Infant formula has been marketed aggressively in developing countries where government capacity to monitor and regulate their marketing is limited [3]. Nepal has adopted the 1981 *‘International Code of Marketing Breastmilk Substitutes’* of the World Health Organization (WHO), and in 1992 passed the ‘*Mother's Milk Substitutes (Control of Sale and Distribution) Act, 2049 (1992)*’. According to this Act, the marketing, advertisement, and the promotion of infant formula is prohibited in Nepal [12]. Within a few years of endorsement of the Act however, the country underwent severe political unrest for more than a decade [13] which may have affected the implementation of the Act. While none of the public hospitals allow the advertising of infant formula on their premises, distribution through pharmacies, grocery shops, and departmental stores is unrestricted. These selling outlets are rapidly proliferating in the urban areas of Nepal.

Infant formula is frequently donated by multinational companies and distributed by humanitarian agencies following natural disasters in developing countries [8]. These donations are often unsolicited and their distribution uncontrolled and widespread [5, 14]. Not only does the uncontrolled distribution of donated infant formula pose a health risk in the immediate aftermath of a natural disaster [5], there is some suggestion that the continued availability of donated formula following the emergency period may undermine traditional breastfeeding practices and contribute to increased rates of prelacteal feeding [8].

To date, few studies have reported on complementary feeding practices in Nepal [15, 16] and none have investigated the factors associated with formula feeding. This study aimed to investigate the prevalence of supplementing breastmilk with infant formula and factors associated with this practice in Western Nepal where the practice of breastfeeding is universal.

## Methods

### Study setting

The study was conducted in the Rupandehi district that is located in the South-western plain area (Terai) of Nepal bordering India. The district has 69 village development committees in rural areas and two municipalities in urban areas which are the lowest administrative units in Nepal. The district has two medical colleges, one zonal hospital (referral hospital), one district hospital, five primary health care centres, six health posts, and 58 sub-health posts [17]. The estimated number of infants in this district for fiscal year 2013/204 was 20,061 (each month: 1672) [Source: District Public Health Office, Rupandehi, Annual Target 2013/2014].

### Study design and sample

A community-based cohort study was conducted between January and October, 2014. A total of 735 (rural 378, urban 357) postpartum mothers who were local residents, had living infants, were within one month postpartum, and had a singleton child, were recruited in the study. The process of participant selection is published in detail elsewhere [18] but briefly participants were recruited from 12 randomly selected communities of urban areas, and 15 village development committees of rural areas. List of eligible participants were prepared with the help of local female community volunteers, and health facilities. The required number of participants was then selected from the list using random sampling. The numbers of mother-infant participants was proportionate to population size based on monthly target of expected number of infants aged <30 days. Participants were recruited from adjacent communities when enough participants could not be recruited from the selected community.

### Instrument and data collection

Face-to-face interviews were conducted by trained female enumerators using structured questionnaires, which were adapted from the Nepal Demographic and Health Survey 2011 [10] and a previous cohort study conducted in central Nepal [19]. The Nepali version of questionnaires was pre-tested among 30 eligible participants to ensure cultural appropriateness before use in this study. Some words were replaced with equivalent local terms however, no significant changes to individual questions were necessary. A 24-hour-recall method was used to collect information on infant feeding practices including formula feeding, during the first (within 30 days), fourth (90–120 days), and sixth month (150–180 days). Prompted responses were collected by reading a list of common food items provided to infants to ensure better recall by mothers.

### Variables

The binary outcome variable of this study was ‘formula feeding’ coded as 1 (provided infant formula) and 0 (not provided infant formula). An infant was considered ‘formula fed’ if their mother reported providing her infant with formula at any one of the three interviews. A number of independent variables identified in the literature as being associated with the introduction of formula were investigated (Table 1). Briefly, ‘maternal occupation’ was categorised as ‘employed’ (salaried job), ‘semi-employed’ (daily wage, small business), and ‘household or agricultural work’ [20]. Ethnicity was categorised based on caste group similarities into ‘advantaged caste groups’ (Brahmin, Chhetri, Newar, Gurung, Jogi, Thakuri), ‘middle caste groups’ (Janjati, non-Janjati and Muslim), and ‘Dalit caste’ (Bishwakarma, Dhawal, Kami, Pariyar, Pasi, Sunar) [21]. ‘Birthweight’ was recorded as a continuous variable and then categorised into ‘low’ (<2500 grams) and ‘average or greater’ (≥2500 grams).

**Table 1:** Characteristics of participants and the practice of formula feeding in Western Nepal, 2014

Factor	Infant ever fed infant formula (*n*= 711)		*p*-value*
	No	Yes	
	*n* (%)	*n* (%)	
Maternal age (years)^a^			0.425
15–19	51 (9.4)	14 (8.3)	
20–29	396 (73.2)	132 (78.1)	
30–45	94 (17.4)	23 (13.6)	
Maternal education			<0.001
No education	152 (28.0)	33 (19.5)	
Primary to lower secondary	201 (371)	37 (21.9)	
Secondary	94 (17.5)	30 (17.8)	
Higher	95 (17.5)	69 (40.8)	
Maternal occupation			<0.001
Employed–salaried job	15 (2.8)	15 (8.9)	
Semi-employed	118 (21.8)	19 (11.2)	
Household or agricultural work	409 (75.5)	135 (79.9)	
Antenatal care (Frequency)^a^			0.250
No visit	14 (2.6)	2 (1.2)	
1–3 visits	123 (22.8)	31 (18.5)	
4 or more visits	403 (74.6)	135 (80.4)	
Place of delivery			0.103
Home	70 (12.9)	14 (8.3)	
Health facility	472 (87.1)	155 (91.7)	
Mode of delivery			<0.001
Vaginal	484 (89.3)	124 (73.4)	
Caesarean	58 (10.7)	45 (26.6)	
Ethnicity			<0.001
Advantaged caste groups	169 (31.2)	96 (56.8)	
Middle caste groups	300 (55.4)	59 (34.9)	
Dalit caste	73 (13.4)	14 (8.3)	
Sex of child			
Male	277 (51.1)	92 (54.4)	
Female	265 (48.9)	77 (45.6)	
Birth order^a^			0.358
First	227 (42.0)	80 (47.3)	
Second or third	246 (45.4)	73 (43.2)	
Fourth or more	68 (12.6)	16 (9.5)	
Birth weight^a^			0.254
Low (<2500g)	72 (14.3)	16 (10.7)	
Average or more (≥2500g)	432 (85.7)	134 (89.3)	
Place of residence			<0.001
Rural	301 (55.5)	57 (33.7)	
Urban	241 (44.5)	112 (66.3)	

^a^missing data present. *chi-square *p*-value

### Statistical analysis

Types of complementary food including infant formula were descriptively reported as frequency and percentage of infants in the first, fourth and sixth months. Factors associated with formula feeding were investigated using chi-square test followed by logistic regression analyses. The backward stepwise process was used in a multiple logistic regression. Analyses were conducted using the Statistical Package for Social Sciences (SPSS, IBM Statistics, Version. 20).

### Ethics

Ethics approval was obtained from the Nepal Health Research Council (773/2014), and the Human Research Ethics Committee (HR 184/2013) at Curtin University, Australia. Mothers provided consent for themselves and their infants. The participants were also advised that they had the right to refuse to participate or to withdraw from the study at any time without prejudice.

## Results

### Characteristics of participants

**Fig. 1:**
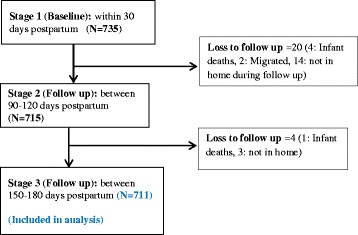
Study interview flow chart

Figure 1 illustrates the interview flow chart of the cohort study that included 735 participants enrolled in the first interview; of which 715 (97.3 %) and 711 (96.7 %) responded to the second and third interviews, respectively. About one in five (22.9 %) of recruited mothers had higher education while only a small proportion (4.1 %) was employed in a salaried job. The majority (76.2 %) of mothers attended four or more antenatal care visits, delivered in health facilities (88.2 %) and 14.1 % delivered via caesarean section. About half (51.4 %) of the participants were from rural areas (Table 1).

### The use of formula feeding as supplementary feeding

**Fig. 2:**
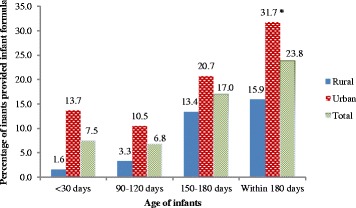
Prevalence of formula feeding among infants upto six months in Western Nepal. *statistically significant

**Fig. 3:**
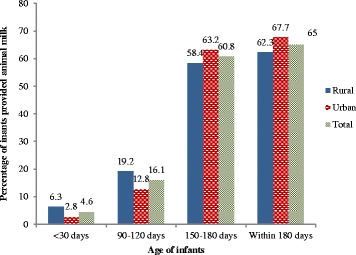
Prevalence of animal milk feeding among infants upto six months in Western Nepal

All mothers were breastfeeding at the time of recruitment. Table 2 presents the types of complementary foods that were provided to infants in the first, fourth and sixth months. In the first month, infant formula was the most common (7.5 %) food given to infants and was used to supplement breast milk. The proportion of infants receiving infant formula increased to 17.0 % in the sixth month (Fig. 2). A total of 169 (23.8 %) of the 711 mothers who completed the third interview reported ‘ever feeding’ infant formula at some time in the first six months. Figure 3 shows the proportion of infants receiving animal milk which was 4.6 % in the first month with a sharp rise in the fourth (16.1 %) and sixth (60.8 %) months.

**Table 2:** Complementary foods introduced during the first six months in Western Nepal, 2014

Complementary foods^a^	First month	Fourth month	Sixth month
	(*N*=735)	(*n*=715)	(*n*=711)
Infant formula	55 (7.5%)	49 (6.8%)	121 (17.0%)
Plain water	24 (3.3%)	203 (28.4%)	485 (68.2%)
Animal milk	34 (4.6%)	115 (16.1%)	432 (60.8%)
Sugar water	0	4 (0.6%)	26 (3.7%)
Sugar salt water	4 (0.5%)	12 (1.7%)	19 (2.7%)
Ghee	12 (1.6%)	26 (3.6%)	26 (3.7%)
Honey	8 (1.1%)	33 (4.6%)	27 (3.8%)
Honey and ghee mixed	2 (0.3%)	16 (2.2%)	20 (2.8%)
Tea	1 (0.1%)	1 (0.1%)	0
Porridge	0	11 (1.5%)	206 (29.0%)
Jaulo Khichadi^b^	0	10 (1.4%)	131 (18.4%)
Adult food	0	2 (0.3%)	27 (3.8%)
Others food items deemed healthy by parents	0	1 (0.1%)	27 (3.8%)
Any complementary feeding	118 (16.1%)	278 (38.9%)	578 (81.3%)

*Note*: dietary recall is based on 24-h recall method. ^a^ multiple response. ^b^ local food that is a mixture of rice, pulses and cereals. It is well cooked to make soft and salt and turmeric are sometime added to improve taste

A total of 55 mothers who were formula feeding their babies at the time of the first interview were asked what brand they were using. Lactogen^©^ (*n* = 16, 29.1 %) and Nestogen^©^ (*n* = 2, 3.6 %) were the two brands most commonly used; the rest of the mothers could not recall the brand name. The brand of formula chosen was based on: hospital advice (*n* = 24, 43.6 %), other people’s advice (*n* = 4, 7.3 %), brand loyalty (*n* = 1, 1.8 %), and advertisement (*n* = 1, 1.8 %). It appeared that formula was mostly provided as top-up food to infants as the methods of formula feeding were reported as: mother’s milk topped-up with formula: (*n* = 29, 52.7 %), half breastmilk and half formula (*n* = 15, 27.3 %) only formula milk (*n* = 3, 5.4 %) and usually formula feeding topped-up with other foods (*n* = 2, 3.6 %).

### Factors associated with formula feeding

Factors associated with formula feeding are presented in Table 3. The results of the stepwise multiple logistic regression indicates that infants who were born to mothers with higher education (adjusted odds ratio (aOR): 2.08; 95 % confidence interval (CI): 1.14 to 3.80), born by caesarean section (aOR: 1.96; 95 % CI: 1.21 to 3.18), and born to families residing in urban areas (aOR: 2.14; 95 % CI: 1.37 to 3.33) were more likely to be formula fed. Figure 2 illustrates that formula feeding prevalence in the first, fourth and sixth months was higher in the urban areas than the rural areas.

**Table 3:** Factors associated with formula feeding in Western Nepal, 2014

Factors	Adjusted odds ratio^a^ (95% CI)	*p*-value^*^
Maternal education		0.007
No education	1.00	
Primary to lower secondary	1.77 (0.79, 3.98)	
Secondary	1.23 (0.65, 2.33)	
Higher	2.08 (1.14, 3.80)	
Mode of delivery		0.006
Vaginal	1.00	
Caesarean	1.96 (1.21, 3.18)	
Place of residence		0.001
Rural	1.00	
Urban	2.14 (1.37, 3.33)	

^a^From stepwise logistic regression model: all variable in Table 1 were included in initial model. ^*^from multiple logistic regression

## Discussion

About one in every four infants was provided infant formula within the first six months of life. An earlier cohort study [9] from Central Nepal reported lower prevalence of formula feeding of 1.7, 6.3 and 13.4 % at 4, 12, and 22 weeks, respectively compared to our findings 7.5, 6.8, and 17.0 %. Another study from the Bhaktapur districts, near the capital city of Nepal reported that 31 % of infants aged nine months were provided powered milk or infant formula [22]. The Government of Nepal has banned the advertising of infant formula in health facilities, and enforced the ‘*Mother's Milk Substitutes (Control of Sale and Distribution) Act, 2049 (1992)*’ [12]. As a result, the provision of infant formula as gifts or free samples is non-existent in public hospitals in Nepal. Similarly, childhood illness management protocols for health workers discourage formula feeding [23]. Despite these efforts, the use of infant formula in Nepal appears to be on the rise; therefore, there is further need to curtail such practice.

In this study, urban mothers were twice as likely as rural mothers to provide formula to their babies. Urban areas have a number of characteristics which make infants particularly vulnerable to formula feeding. The majority of departmental stores and retail pharmacies are located in the urban areas of Nepal, and they sell infant formula without any restriction. Mothers who experience some difficulties in breastfeeding can easily find infant formula in urban areas, and use as an alternative to breastfeeding [24, 25]. Sethi & Mishra [24] reported that the demonstrating of bottles and nipples in pharmacies and grocery stores was used as a form of advertising to promote formula and bottle feeding in India. They argued that the effects of such demonstrations were similar to that of direct television and radio advertising of infant formula. Infant formula displays are commonly found in pharmacy and grocery shops in urban areas of Nepal. We observed the infant formulas displayed in a pharmacy located in front of the entrance of an urban tertiary hospital which is BFHI (baby friendly hospital initiative) accredited, and found that the message on the harmful effect of the use of unboiled water during preparation of infant formula was in English language only and this carries no value in Nepal as the majority of the population cannot understand it due to the language barrier *(Photo not shown due to copyright issue associated with brand name of infant formula)*. Nepal’s public health system is designed with special focus on the rural areas [26]. There are usually inadequate public health networks and health promotion programs in the urban areas. Therefore, urban health programs of Nepal should incorporate breastfeeding programs to help discourage the use of infant formula and counteract the widespread marketing of infant formula in urban areas.

The household financial impact of formula feeding has not been studied before in Nepal. At the time of the study we observed that the price of infant formula ranged 460–480 Nepali Rupees (which was equivalent to 3–4 USD at the time). Given that a quarter of the Nepali population lives on an income of less than1.25 USD per day [27], the expense of 3–4 USD for each formula packet is very costly for the majority of the local population. There is lack of study on the economic impact of formula feeding and further research is needed to provide more information.

Nepal follows the WHO guideline in infant feeding and encourages and promotes exclusive breastfeeding in the first six months of life. When mother’s milk is no longer sufficient due to maternal illness and death, the child health guidelines of the Ministry of Health recommends clean and boiled cow’s milk to avoid contamination and also protect from under nutrition in early infancy [28]. The same recommendation is not given for infant formula for several reasons: (1) likelihood of contamination during preparation, (2) lower literacy rates of mothers and the senior women that is likely to impact their capacity to safely prepare and handle formula milk and (3) poor sanitation status, mainly in rural parts of the country [27]. These factors would lead to an increased rate of childhood diarrhoea which is already one of the leading causes of morbidity and mortality among infants and children of Nepal.

Caesarean delivery was a risk factor for infant formula feeding. A mother who has undergone a caesarean section is often exhausted, usually confined to bed, under the effect of anaesthesia or analgesia, and may suffer anxiety and stress [29]. As a consequence such women are often separated from their newborn or unable to hold and breastfeed their newborn infants, and mothers and families may find it easier to introduce infant formula in such circumstances [30]. Once a newborn is accustomed to infant formula; it is hard to re-establish breastfeeding unless mothers are encouraged and provided support [31]. In addition, the suckling ability of infants delivered by caesarean section tends to be less than their exclusively breastfed counterparts [32]. Mothers, who have undergone caesarean section, are in need of support in the early postnatal period to establish and continue exclusive breastfeeding. When infant formula is the only viable option, for example when the mother is seriously ill or cannot breastfeed, a safe way of preparation should be well communicated, and the harms that can be caused by inappropriately prepared formula feeding must be advised [8]. For example, a guide to the safe use of bottle feeding has been published by the WHO [33] and could be adapted in the Nepalese context.

Higher education of mothers was independently associated with increased likelihood of formula feeding. Highly educated women are also likely to work. While there is a two-month paid maternity leave in the public sector, there is no provision of such leave in the private sector. Thus, these mothers might face difficulties in breastfeeding because of full-time work leaving them with the options to introduce animal milk or infant formula [34, 35].

Within the six months of our data collection, Nepal on 25 April 2015 experienced a devastating earthquake measuring 7.9 on the Richter scale that led to the damage of 750,000 houses, over 8,600 deaths and 17,000 injuries [36]. Hipgrave et al. [5] reported that 80 % of households with children received donated infant formula after a similarly devastating earthquake in Yogyakarta and Central Java, Indonesia in 2006. Follow-up of these children showed that 25.4 % of infants who received donated milk experienced diarrhoea compared with 11.5 % of those infants who did not. Allegedly infant formula donated after the 2015 Nepal earthquake and distributed in Laprak, Gorkha district was date-expired [14] and as such posed a potential health risk. Following natural disasters water supplies are typically contaminated and there is no heat to boil the water and containers [37]. In Nepal, unless strict measures are taken by the Government, the unrestricted sale and distribution of infant formula could result into an increased risk of contamination and childhood diarrhoea and deaths in the post-earthquake period [8, 37].

This study is one of the few longitudinal studies to investigate formula feeding practices in Nepal. An important limitation of this study is that feeding practices were self-reported and may be subject to recall and social desirability bias. However, such self-reported prevalence of infant feeding has been widely used in a number of studies [38–40]. While the short recall time of 24-hour used in cross-sectional studies such as the Demographic and Health Survey is likely to reduce recall bias, there is the possibility of missing the short-term, intermittent use of infant formula resulting in the likelihood of under reporting of prevalence. For instance, the 24 hour recall method, or current status method, has been shown to overestimate the prevalence of exclusive breastfeeding and underestimate the prevalence or partial breastfeeding, when compared to the “since birth” recall method [41]. No attempt was made to collect data on infant illnesses and future studies should focus on reporting additional risk of childhood diarrhoea due to formula feeding.

## Conclusion

This study found that one in every four infant was provided infant formula at some time in their first six months. Infants born to educated mothers, born by caesarean section and born to a family residing in urban areas were more at risk of formula feeding. The ‘*Mother's Milk Substitutes (Control of Sale and Distribution) Act, 2049 (1992)*’ should be strongly enforced, and breastfeeding promotion programs should focus on the mothers with higher education, living in urban areas, and women delivering by caesarean method should be targeted for extra support in the early postnatal period. It should be noted that health promotion programs in the urban areas of Nepal are very limited; therefore, breastfeeding promotion programs should focus on urban areas. Nepal is a country often affected by natural disasters such as landslides and earthquakes; the government should be firm on its position to support breastfeeding and discourage the unrestricted distribution and use of infant formula unless medically indicated.

### Consent for publication

Not applicable

### Availability of data and materials

The datasets supporting the conclusions of this article are available at the institutional repository of Curtin University (http://www.curtin.edu.au). According to the data protection regulation of Curtin University, authors are not permitted to deposit the data elsewhere.
